# Oncologic outcome and potential prognostic factors in primary squamous cell carcinoma of the parotid gland

**DOI:** 10.1186/s12885-019-5969-6

**Published:** 2019-07-31

**Authors:** Qigen Fang, Junfu Wu, Fei Liu

**Affiliations:** 1 Department of Head Neck and Thyroid, Affiliated Cancer Hospital of Zhengzhou University, Henan Cancer Hospital, Zhengzhou, People’s Republic of China; 2 Department of Oral Medicine, The First affiliated hospital of Zhengzhou University, Zhengzhou, People’s Republic of China

**Keywords:** Parotid cancer, Parotid squamous cell carcinoma, Intraparotid node metastasis, Prognosis analysis

## Abstract

**Background:**

Primary parotid squamous cell carcinoma (SCC) is an uncommon tumour, and there is limited data on its prognosis and treatment. The goal of the current study was to analyse the potential prognostic factors and clinical outcomes for this tumour type.

**Methods:**

Consecutive patients with surgically treated primary parotid SCC were retrospectively enrolled in this study. The primary end point was locoregional control (LRC) and disease-specific survival (DSS), which were calculated by the Kaplan-Meier method. Independent prognostic factors were evaluated by the Cox proportional hazards method.

**Results:**

In total, 53 patients were included for analysis. Perineural and lymphovascular invasion were observed in 21 and 16 patients, respectively. Intraparotid node (IPN) metastasis was reported in 23 patients with an incidence rate of 43.3%. Twenty-six patients with cN0 disease underwent neck dissection, and pathologic node metastasis was observed in 10 cases. The 5-year LRC and DS S rates were 35 and 49%, respectively. The Cox model was used to report the independence of disease stage and IPN metastasis in predicting LRC and the independence of disease stage and perineural invasion in predicting DSS.

**Conclusions:**

The prognosis of primary parotid SCC is relatively unfavourable. IPN metastasis significantly decreases disease control, disease stage is the most important prognostic factor, and neck dissection is suggested for patients at any stage.

## Background

Parotid cancers account for 70% of all salivary gland malignancies, and there are 24 different types of malignancies [1–3], of which squamous cell carcinoma (SCC) is one of the least common histologic subtypes. SCC usually conveys a poor 5-year survival rate of less than 50% with an incidence varying from 0.1 to 10% in salivary malignancies [4–10].

Owing to the extreme rarity of parotid SCC, very few authors have focused on this cancer [4–14]. Many studies only describe demographic results, such as parotid SCC being likely to occur in older male patients, but detailed parotid prognostic factors and accurate survival data are unknown [4–7]. All investigators agree that before a diagnosis of primary parotid SCC is confirmed, metastatic SCC from other sites of the head and neck and high grade mucoepidermoid carcinoma must be excluded [4–14]. Recently, two studies were published based on the SEER database. Chen et al. [11] reported that the 5-year DSS rates for patients with stage I, II, III, and IV disease were 86.5, 78.1, 82.4, and 62.8%, respectively, and Pfisterer et al. [12] found that the 5-year DSS rates for patients with stage I, II, III, and IV disease were 80.1, 72.5, 71.3, and 50.3%, respectively. However, these two studies do not specifically identify primary disease from metastatic parotid SCC. Primary parotid SCC might have unique characteristics distinct from metastatic parotid SCC and other parotid cancers. Therefore, the present study aimed to analyse the potential prognostic factors and clinical outcomes of this disease.

## Methods

The Zhengzhou University institutional research committee approved our study, and all participants signed an informed consent agreement for medical research before initial treatment. All experiments were performed in accordance with the Declaration of Helsinki.

From January 2005 to December 2016, medical records of patients with surgically treated parotid SCC were reviewed. Enrolled patients had to meet the following criteria: no previous history of SCC of the head and neck; pathological sections were re-reviewed to exclude the possibility of high grade mucoepidermoid carcinoma with the help of mucin stains [3–9]; and patients had undergone a PET-CT examination to exclude the possibility of metastatic disease. Data regarding age, sex, TNM stage based on the AJCC 7th system, pathological reports, and follow-up were extracted by JF-W and analysed by QG-F, JF-W and FL. Perineural invasion was considered to be present if tumour cells were identified within the perineural space and/or nerve bundle. Lymphovascular infiltration was positive if a tumour was present within the lymphovascular channels [14].

The primary end point was locoregional control (LRC) and disease-specific survival (DSS), which were calculated from the date of surgery to the date of event or latest follow-up, respectively. The Kaplan-Meier approach was used to calculate the LRC and DSS rates, and factors that were significant by univariate analysis were then analysed by the multivariate proportional hazard Cox model to evaluate independent prognostic factors. All statistical analyses were performed by SPSS 20.0, and *p* < 0.05 was considered to be significant.

## Results

In total, 53 (43 male and 10 female) patients with primary parotid SCC were included for analysis. The mean age was 67.4 (range: 32–78) years. The tumour stages were distributed as follows: T1 in 10 cases, T2 in 22 cases, T3 in 13 cases, and T4 in 8 cases. Superficial parotidectomy was performed in 18 patients, and total parotidectomy was performed in 35 cases. Perineural and lymphovascular invasion were observed in 21 and 16 patients, respectively. Intraparotid node (IPN) metastasis was observed in 23 patients with an incidence rate of 43.3%. Negative margins were achieved in 49 patients (Table 1).

**Table 1:** Descriptive characteristics of the enrolled patients

Variables	Number (%)
Sex	
Male	43 (81.1%)
Female	10 (18.9%)
Operation extent	
Superficial parotidectomy	18 (34.0%)
Total parotidectomy	35 (66.0%)
Tumor stage	
T1	10 (18.9%)
T2	22 (41.5%)
T3	13 (24.5%)
T4	8 (15.1%)
Neck lymph node stage	
N0	30 (56.6%)
N+	23 (43.4%)
Perineural invasion	21 (39.6%)
Lymphovascular invasion	16 (30.2%)
Intraparotid node metastasis	23 (43.3%)
Negative margin	49 (92.5%)

In 40 patients with cN0 disease, 26 cases underwent neck dissection of level I-III, pathological neck metastasis was reported in 10 cases (Table 2), and extracapsular spread was observed in 3 cases. Thirteen patients with cN+ disease underwent neck dissection of level I-V, and pathological neck metastasis was reported in all cases, with extracapsular spread observed in 7 cases.

**Table 2:** Distribution of clinical stages in patients undergoing neck dissection for cN0 disease

	cN0 (*n* = 26)			
	cT1	cT2	cT3	cT4
pN0	4	8	2	2
pN+	2	3	2	3

The mean follow-up time was 67.3 (range: 4–135) months, 45 patients received adjuvant radiotherapy, and 16 cases underwent adjuvant chemotherapy. Locoregional recurrence was observed in 35 patients: 10 cases locally, 14 cases regionally, and 11 cases with simultaneous local and regional recurrence. Fourteen patients received salvage surgery, and 21 patients underwent palliative radiochemotherapy. Chemotherapy regimens were primarily based on docetaxel in combination with cisplatin.

**Fig. 1:**
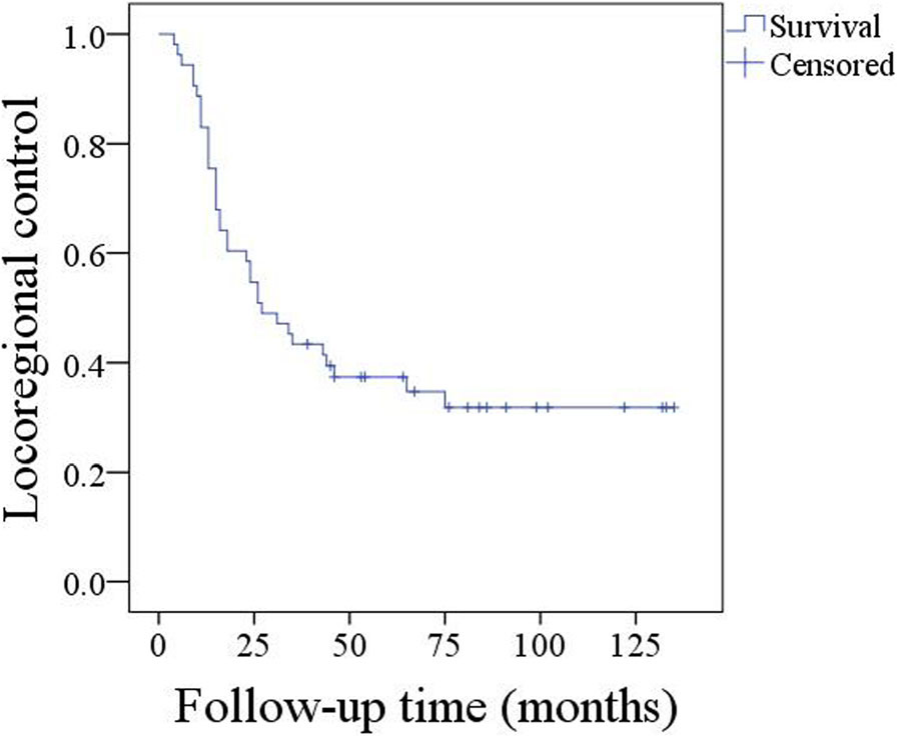
Locoregional control survival in patients with primary parotid squamous cell carcinoma

The 5-year LRC rate was 35% (95%CI: 24–46%) (Fig. 1), and most (68.6%, 24/35) recurrence occurred within 2 years after surgery. Univariate analysis (log-rank test) revealed that an advanced disease stage, extracapsular spread, and IPN were associated with decreased LRC. Further Cox modelling reported the independence of disease stage (*p* < 0.001, 4.122[1.578–16.142]) and IPN (*p* = 0.007, 2.347[1.279–5.612]) in predicting LRC (Table 3).

**Table 3:** Prognostic factors for locoregional control in patients with primary parotid squamous cell carcinoma

	Univariate analysis	Cox model	
	Log rank test	HR[95%CI]	*p*
Age (< 67 vs ≥67)	0.541		
Sex (Male vs female)	0.194		
Tumor stage (T1 + T2 vs T3 + T4)	0.188		
Node stage (N0 vs N+)	0.097		
Disease stage (I + II vs III + IV)	0.008	4.122[1.578–16.142]	< 0.001
Surgery (TP vs SP)^a^	0.333		
Perineural invasion	0.256		
Lymphovascular invasion	0.142		
Intraparotid node metastasis	0.027	2.347[1.279–5.612]	0.007
Margin status	0.679		
Adjuvant radiotherapy	0.147		
Adjuvant chemotherapy	0.633		
Extracapsular spread	0.014	3.841[0.946–8.445]	0.067

^a^: *TP* Total parotidectomy, *SP* Superficial parotidectomy

**Fig. 2:**
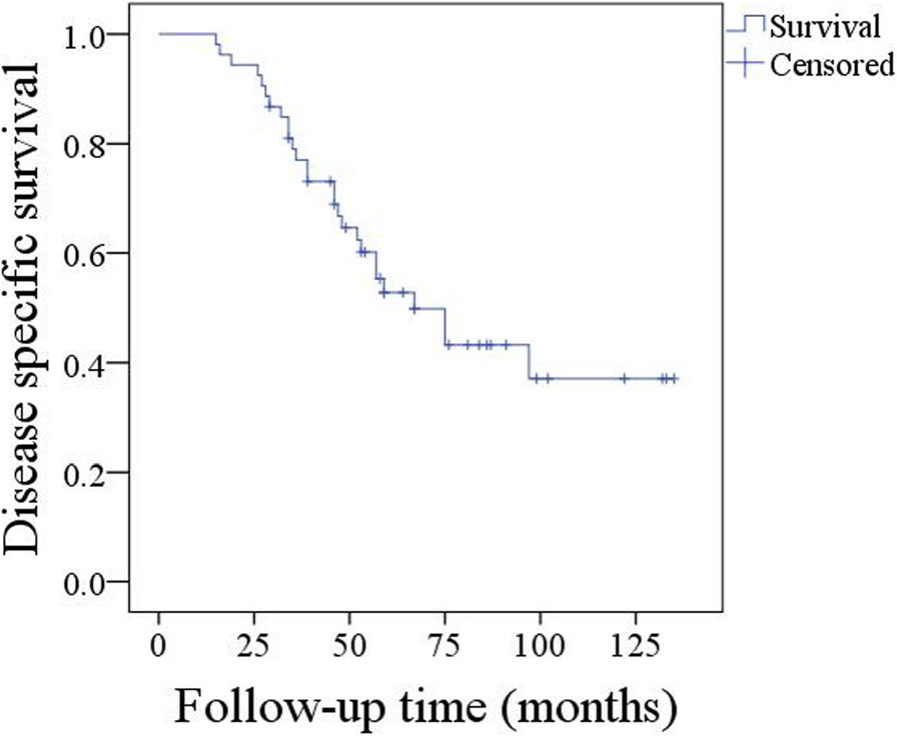
Disease specific survival in patients with primary parotid squamous cell carcinoma

A total of 27 patients died from the disease, and the 5-year DSS was 49% (95%CI: 38–60%) (Fig. 2). Univariate analysis (log-rank test) revealed an advanced disease stage, perineural invasion, and high tumour stage were associated with decreased DSS. Further Cox modelling revealed the independence of disease stage (*p* < 0.001, 5.956[1.875–17.324]) and perineural invasion (*p* = 0.004, 2.113[1.278–7.645]) in predicting DSS (Table 4).

**Table 4:** Prognostic factors for disease specific survival in patients with primary parotid squamous cell carcinoma

	Univariate analysis	Cox model	
	Log rank test	HR[95%CI]	*p*
Age (< 67 vs ≥67)	0.845		
Sex (Male vs female)	0.416		
Tumor stage (T1 + T2 vs T3 + T4)	0.031	2.645[0.745–13.241]	0.144
Node stage (N0 vs N+)	0.154		
Disease stage (I + II vs III + IV)	0.007	5.956[1.875–17.324]	< 0.001
Surgery (TP vs SP)^a^	0.362		
Perineural invasion	0.019	2.113[1.278–7.645]	0.004
Lymphovascular invasion	0.225		
Intraparotid node metastasis	0.411		
Margin status	0.632		
Adjuvant radiotherapy	0.522		
Adjuvant chemotherapy	0.146		
Extracapsular spread	0.287		

^a^: *TP* Total parotidectomy, *SP* Superficial parotidectomy

## Discussion

Primary parotid SCC has an aggressive clinical presentation, and most patients experienced disease recurrence within 2 years after the initial treatment. The 5-year LRC and DSS rates in the current study were only 35 and 49%, respectively. Flynn et al. [4] reported the oncologic outcome of 8 cases, reporting that seven patients died of the disease, with only 1 case being alive and free of disease. Lee et al. [5] retrospectively enrolled 12 patients with primary parotid SCC and described the regional and local failure rates as 25 and 58%, respectively. Similar findings were noted by Sterman et al. [6] and Gaughan et al. [7]. However, these studies were limited by a relatively small sample size. Recently, two studies were published with large sample sizes focusing on parotid SCC based on the SEER database. Chen et al. [11] reported that the 5-year DSS rates for patients with stage I, II, III, and IV disease were 86.5, 78.1, 82.4, and 62.8%, respectively, and Pfisterer et al. [12] found that the 5-year DSS rates for patients with stage I, II, III, and IV disease were 80.1, 72.5, 71.3, and 50.3%, respectively. These survival data are slightly better than those reported in the present study, but these two studies did not specifically identify primary disease from metastatic parotid SCC. Our outcome is more consistent with a report by Wang et al. [10], wherein 20 of 34 patients developed disease recurrence and 19 patients died, with a 5-year DSS rate of 50.3%.

Perineural and lymphovascular invasion, as well as extracapsular spread, are well known adverse pathological characteristics [15–18]. Walvekar et al. [7] reported that high grade parotid cancer is more likely to exhibit extracapsular spread. Lee et al. [18] described that compared to low grade parotid cancers, perineural and lymphovascular invasion were more common in high grade malignancies. SCC is recognized as one kind of high grade parotid malignancy, but no studies have reported the detailed frequency of such adverse pathological characteristics. Unlike parotid adenocarcinoma, parotid SCC might have unique characteristics, including a higher likelihood of perineural and lymphovascular invasion. Our findings support this hypothesis, and incidences were slightly higher than those previously reported [19–21].

Neck lymph node metastasis is an important prognostic factor in head and neck cancer [22–25], but the role of elective neck dissection on cN0 parotid cancer remains controversial. A common principle is that N0 necks should be electively treated when the occult metastatic rate is greater than 20% [26]. Ali et al. [27] studied 263 patients at Memorial Sloan-Kettering Cancer Center and concluded that in patients with cN0 disease, observation of the neck was safe in those under 60 years of age with clinical T1 or T2 tumours who had low-grade histology. END should be performed in patients with cT3T4 disease or high-grade histology and should involve levels II to IV at a minimum. Armstrong et al. [28] retrospectively reported that overall occult lymph node metastasis occurred in 12% of 474 salivary gland cancer patients, and multivariate analysis demonstrated a positive association between pathological tumour grade and risk of occult metastasis. The authors subsequently concluded that END should be reserved for high grade tumours and for those with larger primary tumours. This viewpoint was also supported by other investigators [29, 30]. In the current study, we found that the rate of occult neck metastasis was 27.8% for early stage parotid SCC, while previous authors described a variable occult metastasis rate from 41 to 60% [8]. These findings all suggest the necessity of routine neck dissection for treating primary parotid SCC, even in early stage disease.

The prognostic factors for parotid cancer have been widely analysed. Accepted survival predictors include high tumour stage, neck lymph node metastasis, perineural invasion, lymphovascular invasion, pathological tumour grade, neutrophil-to-lymphocyte ratio, resection margin, and intraparotid node metastasis [1, 3, 22–24]. Niu et al. [23] retrospectively enrolled 35 patients with sarcomatoid carcinoma in the parotid gland, concluding that perineural invasion was the most important predictive factor. Chang et al. [24] analysed the oncologic outcome in 98 patients with primary parotid cancer and found that the pathological T stage, resection margin, external parenchymal extension, pathological lymph node status, and maximum standardized uptake value were significantly related to DSS by univariate analysis. Further Cox modelling revealed that the pathological lymph node status and maximum standardized uptake values were independent prognostic factors. Similar findings were also observed in the current study. However, we noted that there was a relatively high rate of IPN metastasis and that, interestingly, IPN metastasis was associated with a higher recurrence risk. The association between IPN metastasis and prognosis in parotid cancer has rarely been evaluated. Lim et al. [25] simply described that IPN metastasis was related to poor disease control. Feng et al. [19] previously reported that IPN metastasis was associated with poor local control and that metastatic IPNs conveyed a worse prognosis. A possible explanation might be that there are lymph nodes in both lobes of the parotid gland and that positive deep lymph nodes might remain after superficial or lateral parotidectomy. Furthermore, residual disease might be present, and recurrent disease was expected.

Key limitations in this study must be acknowledged. Although this population was representative, it is difficult to draw firm conclusions regarding the clinical importance of IPN metastasis and routine neck dissection. However, these findings indicated that the accurate role of IPN metastasis in prognosis deserves additional and larger prospective studies. Second, the sample size was relatively small, and additional large sample sizes or multicentre studies are needed to clarify these questions.

## Conclusions

In summary, the prognosis of primary parotid SCC is relatively unfavourable, and IPN metastasis significantly decreases disease control. Furthermore, disease stage is the most important prognostic factor, and neck dissection is suggested for any stage patient.

## Data Availability

All data generated or analysed during this study are included in this published article. The primary data can be received from the corresponding author.

## Abbreviations

DSS: Disease-specific survival
IPN: Intraparotid node
LRC: Locoregional control
SCC: Squamous cell carcinoma
